# Comparative In Silico Analysis of Ultra-Hypofractionated Intensity-Modulated Photon Radiotherapy (IMRT) Versus Intensity-Modulated Proton Therapy (IMPT) in the Pre-Operative Treatment of Retroperitoneal Sarcoma

**DOI:** 10.3390/cancers15133482

**Published:** 2023-07-04

**Authors:** Emile Gogineni, Hao Chen, Alexandra C. Istl, Fabian M. Johnston, Amol Narang, Curtiland Deville

**Affiliations:** 1 Department of Radiation Oncology, The Ohio State University Wexner Medical Center, Columbus, OH 43210, USA; 2 Department of Radiation Oncology and Molecular Radiation Sciences, Johns Hopkins University School of Medicine, Baltimore, MD 21231, USA; hchen142@jhmi.edu (H.C.); anarang2@jhmi.edu (A.N.); cdeville@jhmi.edu (C.D.J.); 3 Department of Surgical Oncology, Medical College of Wisconsin, Milwaukee, WI 53226, USA; aistl@mcw.edu; 4 Department of Surgery, Johns Hopkins University School of Medicine, Baltimore, MD 21287, USA; fjohnst4@jhmi.edu

**Keywords:** retroperitoneal sarcoma, preoperative, hypofractionated, ultrahypofractionated, radiation, proton therapy, particle therapy, IMPT, intensity-modulated, dosimetric

## Abstract

**Simple Summary:**

While pre-operative radiation did not improve abdominal recurrence-free survival for retroperitoneal sarcoma in the randomized STRASS trial, it did reduce rates of local recurrence. However, the risk of toxicity was substantial and the time to surgery was prolonged. A combination of hypofractionation and proton therapy may reduce delays from the initiation of radiation to surgery and limit the dose to surrounding organs at risk. We conducted a dosimetric comparison of the pre-operative ultra-hypofractionated intensity-modulated photon radiotherapy and proton therapy using a five-fraction regimen of 25 Gy radiobiological equivalent (GyE) to the clinical target volume and 30 GyE to the margin-at-risk (radiobiological effective dose 1.1). Proton therapy maintained target coverage while significantly reducing the dose to adjacent organs at risk and the integral dose compared to photons. Further investigation is warranted to validate these dosimetric findings and potential clinical benefit. A prospective trial treating retroperitoneal sarcoma with pre-operative ultra-hypofractionated proton therapy at our institution is currently being pursued.

**Abstract:**

Background: While pre-operative radiation did not improve abdominal recurrence-free survival for retroperitoneal sarcoma (RPS) in the randomized STRASS trial, it did reduce rates of local recurrence. However, the risk of toxicity was substantial and the time to surgery was prolonged. A combination of hypofractionation and proton therapy may reduce delays from the initiation of radiation to surgery and limit the dose to surrounding organs at risk (OARs). We conducted a dosimetric comparison of the pre-operative ultra-hypofractionated intensity-modulated photon (IMRT) and proton radiotherapy (IMPT). Methods: Pre-operative IMRT and IMPT plans were generated on 10 RPS patients. The prescription was 25 Gy radiobiological equivalents (GyEs) (radiobiological effective dose of 1.1) to the clinical target volume and 30 GyEs to the margin at risk, all in five fractions. Comparisons were made using student T-tests. Results: The following endpoints were significantly lower with IMPT than with IMRT: mean doses to liver, bone, and all genitourinary and gastrointestinal OARs; bowel, kidney, and bone V5–V20; stomach V15; liver V5; maximum doses to stomach, spinal canal, and body; and whole-body integral dose. Conclusions: IMPT maintained target coverage while significantly reducing the dose to adjacent OARs and integral dose compared to IMRT. A prospective trial treating RPS with pre-operative ultra-hypofractionated IMPT at our institution is currently being pursued.

## 1. Introduction

Sarcoma represents an uncommon group of malignancies, accounting for less than 1% of malignant adult tumors, with 15% of these originating in the retroperitoneum [1,2]. Multidisciplinary evaluation by clinicians experienced in the treatment of this rare malignancy is vital to provide acceptable outcomes [1]. Surgery is the standard of care for all retroperitoneal sarcomas (RPSs); however, a 25–50% rate of local recurrence after surgery alone remains high [3,4,5,6,7]. Additionally, rates of microscopic and macroscopic positive margins are 12–58% and 20–25%, respectively, with positive margins correlating with poor control [3,8,9,10,11,12,13,14,15,16,17,18,19].

Radiation therapy (RT) is often delivered preoperatively in order to decrease the risk of local recurrence. Due to the large volume of RPS tumors, with median sizes of 17–18 cm at presentation, and the close proximity of surrounding normal tissue, the risk of RT-associated toxicity is substantial, with >30% rates of grade ≥ 3 toxicity [3,4,20,21]. Controversy around RT for RPS persists after the completion of STRASS, the only phase III randomized trial exploring outcomes after pre-operative RT for RPS [7]. Despite being a negative study for its primary endpoint of abdominal recurrence-free survival, STRASS yielded results that give nuance to our path forward in the study of RPS: the local recurrence rate was 28% in irradiated patients versus 64% in those who underwent surgery alone, and post hoc subgroup analyses suggested that RT may provide greater benefit in liposarcoma (the most common RPS histology, representing 75% of subjects in this trial). Furthermore, STRASS drew attention to patterns of failure, delays to surgery, and the significance of RT-associated toxicity. This trial and other studies suggest that local recurrence accounts for 75% of cancer-related mortality in RPS [5,6] and that margin positivity is associated with worse survival [3,8,9,10,11,12,13,14,15,16,17,18,19].

Progression during RT also presents a significant issue, as it represents the most common event contributing to abdominal recurrence in STRASS as assessed by Response Evaluation Criteria in Solid Tumors (RECIST). Correspondingly, many clinicians are hesitant to delay curative surgery for weeks to months to deliver pre-operative RT without supporting prospective evidence. Thus, finding methods to mitigate the issues of delaying surgery and irradiating normal tissue may provide a path towards affording the benefits of RT while limiting its downside.

Hypofractionation represents one potential solution to avoid surgery delays and minimize the risk of progression during RT. In addition to the convenience for patients and clinical workflow, a low α/β ratio of sarcomas suggests that hypofractionation may also provide radiobiological benefits [22,23]. While five fraction ultra-hypofractionated regimens are becoming increasingly utilized for extremity and truncal sarcomas, the literature addressing its use for RPS is limited to small subsets of retrospective studies [24,25,26,27,28,29,30]. This is likely due to the risk of acute and late toxicity when using high doses per fraction for large tumors surrounded by radiosensitive organs at risk (OARs). Correspondingly, hypofractionation is not discussed as an option in current RPS guidelines published by Baldini et al. in 2015 and Salerno et al. in 2021 [31,32].

Proton therapy lacks the exit dose and integral exposure associated with photon irradiation, improving its therapeutic ratio [33], and serves as an option to limit OAR dose and potentially reduce the risk of toxicity with ultra-hypofractionation. A previous phase I/II trial demonstrated the safety and feasibility of conventionally fractionated proton therapy for RPS in the pre-operative setting [34].

In this study, we performed a dosimetric comparison between ultra-hypofractionated intensity-modulated photon radiotherapy (IMRT) and modern scanning beam intensity-modulated proton therapy (IMPT) for the pre-operative treatment of RPS. We hypothesized that IMPT would significantly reduce the dose to the bowel, liver, and kidneys without sacrificing clinical target volume (CTV) coverage.

## 2. Materials and Methods

Following institutional review board approval, our prospectively maintained institutional database was queried to identify 10 patients with RPS previously treated with conventionally fractionated pre-operative photon irradiation. Patients with recurrent tumors and distant metastases and those who had received previous RT were excluded from analysis.

### 2.1. Simulation

Patients were simulated using four-dimensional (4D) computerized tomography (CT) scans in the supine position with arms above their head using vac loc for immobilization with and without IV contrast. Magnetic resonance imaging (MRI) and/or positron emission tomography (PET) images were fused when available. Patients with tumors in close proximity to the stomach were instructed to be NPO (no oral intake of food or liquid) 3–5 h before the simulation.

### 2.2. Volume Delineation

All CTV and OAR contours were delineated by an attending radiation oncologist in the RayStation treatment planning system (RaySearch Laboratories, Stockholm, Sweden) in accordance with published guidelines [31,32]. The gross tumor volume (GTV) was defined as all areas of gross tumor seen on imaging. The iGTV included gross tumor in all phases of breathing, including maximal intensity projection, end inspiration, and end expiration. CTVs were created using a 1.5 cm isometric expansion from the iGTV, respecting the barriers of spread. When the tumor extended to the inguinal canal, CTV margins were expanded to 3.0 cm inferiorly. An additional CTV Boost was delineated according to published guidelines, defined as the area at risk for a positive margin [35]. Planning target volumes (PTVs) were generated for the purposes of IMRT planning using 0.5 cm isometric expansions from CTVs, cropped 0.3 cm from the skin.

Contoured OARs included the stomach, duodenum, bowel, liver, ipsilateral and contralateral kidneys, ipsilateral and contralateral femoral heads, bone, spinal canal, skin, and body. The OAR ‘Bone’ included all bones 1.0 cm cranial and caudal to the CTV and was created as a surrogate for the dose to the bone marrow. The OAR ‘Body—CTV’ included the dose to the entire patient excluding any overlapping CTV.

### 2.3. Treatment Planning

The prescription was 25 Gy radiobiological equivalent (GyE) to the entire CTV and 30 GyE to CTV Boost, all in five fractions. Proton therapy doses were calculated using a radiobiological effective (RBE) dose of 1.1.

IMRT and IMPT plans were created for each patient with predefined CTV and OAR dose-volume histogram (DVH) objectives as outlined in Table 1, Table 2 and Table 3. Table 1 and Table 2 were created from internal institutional directives. Planning objectives listed in Table 3 were derived from 5-fraction biologically effective dose (BED) equivalents of those in published guidelines, assuming an α/β of 3.0 for all OARs [31].

**Figure 1:**
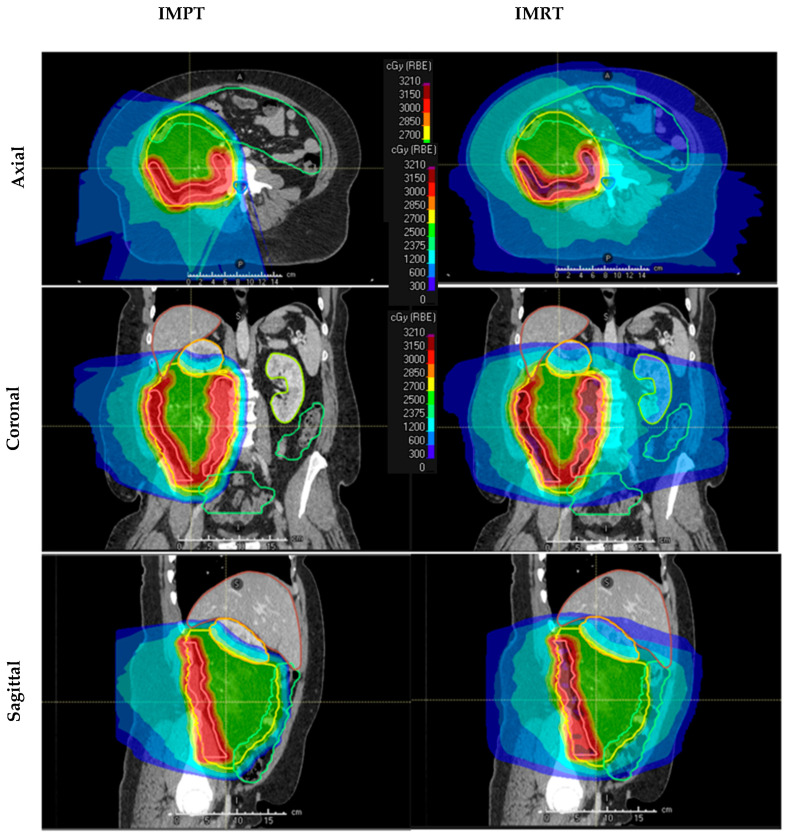
Representative dose-color wash depicting the intensity-modulated proton therapy (IMPT—left) and intensity-modulated photon radiotherapy (IMRT—right) plans in axial (top), coronal (middle), and sagittal (bottom) views. As shown, IMPT spared the low-dose bath to the surrounding organs at risk, which was associated with IMRT plans, particularly the bowel, kidneys, and bone.

IMRT plans were generated using volumetric modulated arc therapy (VMAT), consisting of six 360-degree arcs with 6 collimator rotation angles. IMPT plans were created using 4–5 lateral oblique and/or posterior oblique beams with proton beam angles selected to avoid the couch edge and bowel. An example of the dose-color-wash distribution of each plan can be seen in Figure 1.

All plans were generated using the RayStation treatment planning system. The proton plans consisted of IMPT using pencil beam scanning with discrete spot scanning. Inverse optimization was used to generate appropriate dose distribution with a pre-specified weighting of target coverage and OAR sparing using modulation of beam spot location, energy, and weight. Plans were optimized with 80% single-field optimization (SFO) and 20% multi-field optimization (MFO), prioritizing sparing uninvolved kidney(s) and the spinal canal. All proton plan optimization combined expansion and robust optimization with 3.5% range uncertainty and 0.5 cm setup uncertainty from CTV.

### 2.4. Statistical Analysis

The volumetric percentage of GTV, CTV, CTV Boost, PTV, PTV Boost, stomach, duodenum, bowel, liver, ipsilateral, contralateral, and bilateral kidneys, each femoral head, and bone along the entire DVH were evaluated. Comparative maximum doses to the stomach, duodenum, bowel, each femoral head, spinal canal, and body, in addition to mean doses for all OARs, were assessed. Integral dose to Body—CTV was also captured. Student t-tests were used to compare the plans, with *p* < 0.050 considered statistically significant. All statistical analyses were conducted using Matlab Version R2023a (MathWorks, Natick, MA, USA) and Excel Version 16.74 (Microsoft, Redmond, WA, USA).

## 3. Results

**Figure 2:**
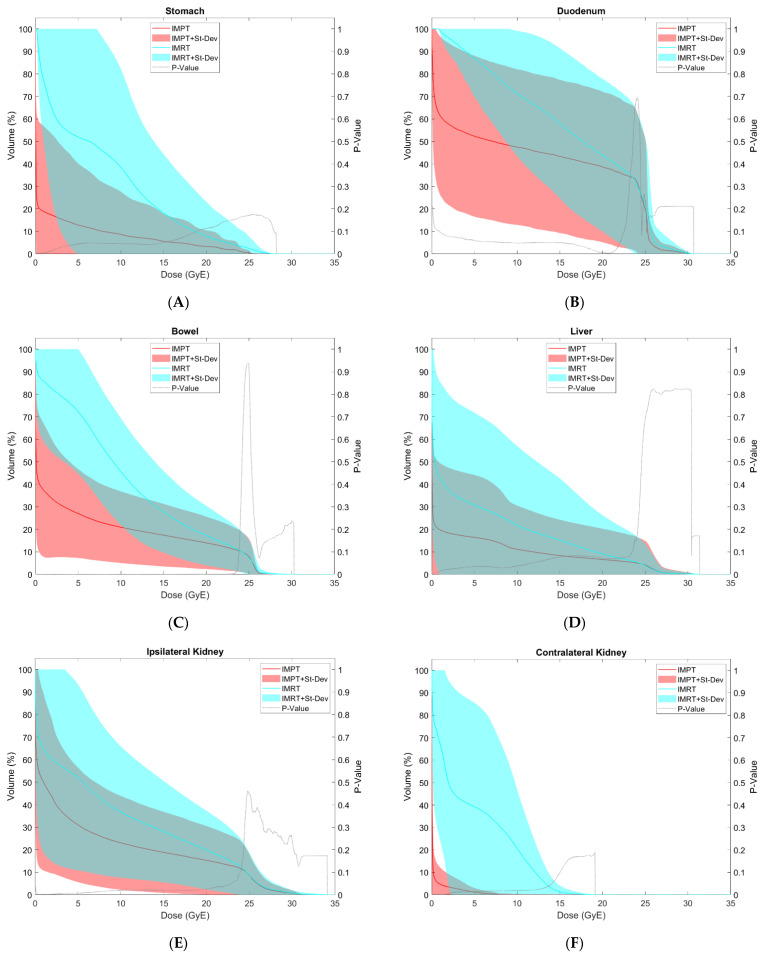

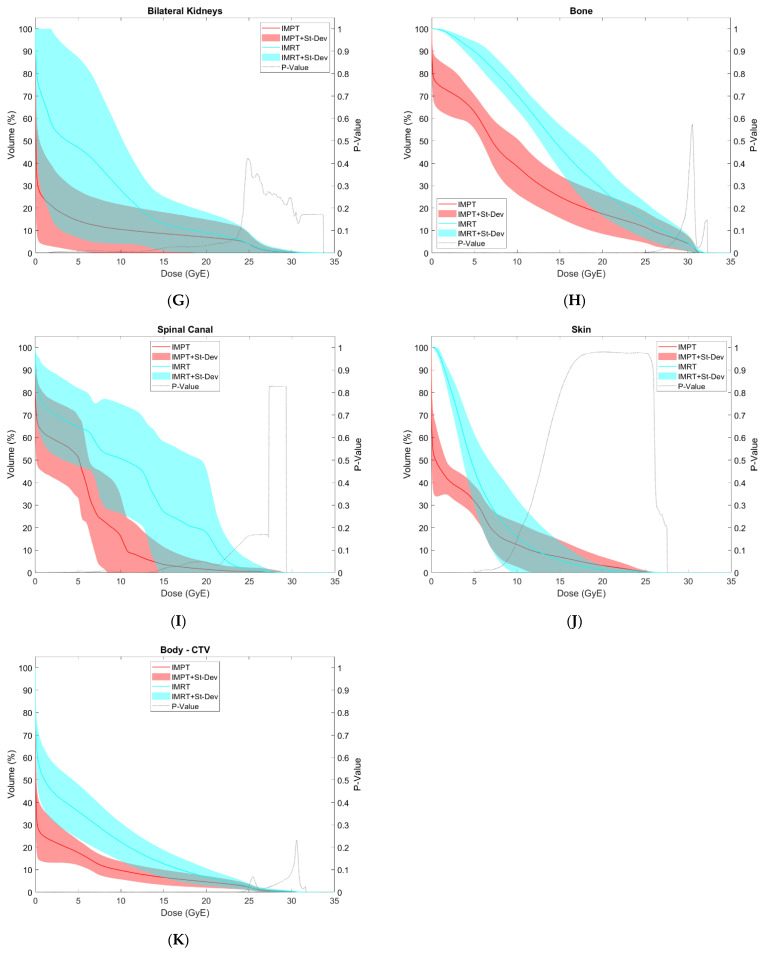
(**A**–**K**). Doses to each organ at risk (OAR) shown over the entire dose–volume histogram comparing intensity-modulated proton therapy (IMPT—red) and intensity-modulated photon radiotherapy (IMRT—blue), including stomach (**A**), duodenum (**B**), bowel (**C**), liver (**D**), ipsilateral kidney (**E**), contralateral kidney (**F**), bilateral kidneys (**G**), bone (**H**), spinal canal (**I**), and body—CTV (**K**). IMPT plans provided significantly lower volumetric endpoints to several OARs, particularly the bowel, kidney, and bone, in addition to providing significantly lower mean doses to nearly all OARs and the whole-body integral dose.

Table 4 provides comparative mean values for target coverage and doses to OARs for IMRT and IMPT plans. Comparative DVHs for OARs are shown in Figure 2A–K.

### 3.1. Target Coverage

CTV coverage was met for IMRT and IMPT plans, with >99% of CTVs receiving ≥100% of the prescription doses for both CTV and CTV Boost. PTV V25 was significantly lower with IMRT than with IMPT. There were no other significant differences in target coverage, and all GTV and CTV planning goals were met with IMRT and IMPT plans.

### 3.2. OAR Comparison

IMRT plans exceeded constraints for bowel V10.45 and for ipsilateral kidney V12, while IMPT plans met these constraints. IMRT and IMPT plans met all other OAR constraints. Dose to OARs was numerically higher with IMRT than IMPT for all analyzed endpoints apart from bowel V25 and ipsilateral femoral head V30, which were higher with IMPT (not statistically significant).

The following dosimetric OAR endpoints were significantly higher with IMRT than IMPT plans: stomach V15, D50%, maximum dose (Figure 2A); bowel V5, V10, V15, V20, D50% (Figure 2C); liver V5, D50% (Figure 2D); ipsilateral, contralateral, and bilateral kidneys V10, V12, D50% (Figure 2E–G); ipsilateral femoral head V23.2; bone V5, V10, V15, V20, V25 (Figure 2H); spinal canal maximum dose (Figure 2I); and body maximum dose. Other dosimetric endpoints which were numerically higher with IMRT than IMPT did not reach statistical significance, with *p*-values 0.051–0.095: stomach V5, V10, V20 (Figure 2A); duodenum V5, V10, V15, V20, D50% (Figure 2B); bowel V25 (Figure 2C); liver V10 (Figure 2D); ipsilateral and contralateral femoral head maximum doses.

Mean doses for nearly all OARs analyzed were significantly higher with IMRT than with IMPT, including stomach, duodenum, bowel, liver, ipsilateral, contralateral, and bilateral kidneys, and bone. Ipsilateral and contralateral femoral head mean and maximum doses were numerically higher with IMRT than with IMPT, but did not reach statistical significance (*p*-values 0.050–0.092). Integral dose to body—CTV was also significantly higher with IMRT than with IMPT (Figure 2K). No OAR endpoint was significantly lower with IMRT than with IMPT plans.

## 4. Discussion

The results from this study show that IMPT provides comparable target coverage to IMRT when treating RPS with ultra-hypofractionated pre-operative radiation while significantly reducing doses to adjacent OARs. We found that several dosimetric endpoints were significantly lower with IMPT, including mean doses to liver, bone, and all genitourinary and gastrointestinal (GI) OARs; volumes of bowel, kidney, and bone receiving doses between 5 and 20 GyE; maximum doses to the stomach, spinal canal, and body; and the whole-body integral dose. While others have compared photon and proton radiation for RPS using conventional fractionation [36,37,38], this represents the first dosimetric study evaluating pre-operative ultra-hypofractionated radiation for retroperitoneal sarcoma.

### 4.1. Correlation between Dosimetry and Toxicity

While our study did not report toxicity outcomes, several dosimetric parameters have been found to correlate with toxicity. A study by Mak et al. evaluating conventionally fractionated pre-operative RT for RPS found that volumes of bowel receiving doses between 10 and 50 Gy correlated with grade ≥2 GI toxicity, with V30 (BED equivalent of ~18 Gy over five fractions with α/β 3.0) being the best discriminator for toxicity [39]. In our study, volumes of irradiated bowel were significantly lower with IMPT for doses between 5 and 20 GyE (see Table 4 and Figure 2C), suggesting this may correlate with lower rates of GI toxicity.

Studies evaluating outcomes for other abdominopelvic organs have shown a similar correlation between bowel dose and GI toxicity, such as Banerjee et al., who showed volumes of bowel receiving doses between 15 and 25 Gy via conventional fractionation predicts toxicity [40]. They proposed a bowel constraint of V15 < 830 cc. BED equivalent doses of 15 Gy for ultra-hypofractionation (10.45 Gy) were significantly higher with IMRT than with IMPT in our study, and IMPT plans achieved the equivalent constraint of V10.45 < 830 cc, while IMRT plans did not.

Ipsilateral femoral head V23.2 was significantly higher with IMRT than with IMPT. V40 has been shown to correlate with the risk of osteoarthritis when using conventional fractionation, correlating with a BED equivalent of V23.2 for the ultra-hypofractionated regimen used in this study [41]. Another metric worth highlighting is the significantly lower doses to the bone for all analyzed endpoints (see Table 4 and Figure 2H). This is particularly noteworthy given the high rates of hematologic toxicity in the STRASS trial, with 77% of patients experiencing grade ≥3 lymphopenia [7]. When a dose to the bone is used as a surrogate for bone marrow, it is possible that IMPT may provide lower rates of lymphopenia and hematologic toxicity than IMRT in this setting.

### 4.2. Dosimetric Correlates for Toxicity with Ultra-Hypofractionation

While there are limited data investigating the use of ultra-hypofractionated RT for RPS, several studies have reported outcomes of hypofractionated RT and stereotactic body radiation therapy (SBRT) for pancreatic cancer and other abdominopelvic malignancies. Bae et al. analyzed variables associated with severe GI toxicity in patients receiving SBRT for abdominopelvic malignancies [42]. They found bowel V20 to be the best dosimetric predictor of toxicity, which was significantly higher with IMRT than with IMPT in our study (see Table 4 and Figure 2C).

Tseng et al. conducted an exploratory analysis of a prospective trial in which patients with pancreatic cancer were treated with hypofractionated proton therapy in doses of 25 GyE in five fractions [43]. Several dosimetric parameters were associated with an increased risk of nausea or vomiting, including stomach V5, V10, V15, and mean dose. All of these endpoints were higher with IMRT than IMPT plans in our study, with *p*-values between 0.019 and 0.056 (see Table 4 and Figure 2A), suggesting IMPT may limit acute GI toxicity in this setting. Several trials evaluating the use of SBRT for pancreatic cancer have used a stomach constraint of V12 < 50 cc, such as ABC-07 and SPARC [44,45]. The American Society for Radiation Oncology (ASTRO) clinical practice guidelines for liver SBRT proposed a stomach constraint of V18 < 10 cc when treating with five fractions [46]. IMPT plans in our study achieved these goals, while IMRT plans exceeded both proposed constraints.

A Phase I trial investigating the use of five-fraction photon RT for pancreatic cancer conducted by Koong et al. showed low rates of acute GI toxicity [47]. They listed mean doses to 50% of each OAR, providing volumetric constraints that may limit the risk of GI toxicity when achieved. IMPT plans in our study met these endpoints for the duodenum D50% 13.2 GyE (<14.5 Gy), left kidney D50% 0.0 GyE (<1.5 Gy), and right kidney D50% 3.4 GyE (<2.0 Gy). Meanwhile, IMRT plans met none of these endpoints, with significantly higher D50%s than IMPT plans for stomach, bowel, liver, and kidneys.

IMRT plans in our study also exceeded our ipsilateral kidney constraint of V12 Gy < 33%. Other studies have proposed more conservative V12 < 25% constraints [48]. IMPT plans achieved both constraints with significantly lower doses to ipsilateral, contralateral, and bilateral kidneys compared to IMRT. This suggests a lower long-term risk of renal toxicity with IMPT, an important consideration in RPS given the frequency of ipsilateral nephrectomy at the time of surgery.

### 4.3. Literature Reporting Outcomes with Proton Therapy for RPS

Limited data exist evaluating clinical outcomes of patients with RPS treated with protons outside of small retrospective series [49,50,51] and a single prospective dose escalation trial [34], all of which used conventional fractionation. However, published guidelines state that proton therapy is acceptable for RPS at experienced centers [31].

In a Phase I dose escalation trial by Delaney et al., IMPT was used to treat RPS preoperatively with conventional fractionation to doses of 50.4 GyE to the entire volume and a 60.2–63.0 GyE simultaneous integrated boost to the margin at risk [34]. IMPT was well-tolerated, with no patient experiencing dose-limiting toxicity. This contrasts sharply with studies evaluating photon IMRT for RPS, which have reported rates of 7–12% grade 3 GI toxicity and up to 31% overall grade 3 toxicity [7,20,21].

### 4.4. Secondary Malignancy

One additional benefit that proton therapy may provide is a reduction in the risk of secondary malignancy. Xiang et al. compared rates of second cancers among >450,000 patients using the National Cancer Database after primary treatment with photons using 3D and IMRT techniques and with protons [52]. They found that the risk of second cancers was lower with proton therapy (adjusted odds ratio, 0.31; 95% confidence interval, 0.26–0.36; *p* < 0.0001). Our data show a >50% relative reduction in whole-body integral dose with IMPT (see Table 4 and Figure 2K). This reduction is similar to that reported by Swanson et al. in their comparison of conventionally fractionated IMRT and protons for RPS [37].

### 4.5. Limitations and Future Direction

This study is limited by its retrospective design and therefore inherent biases that affect all retrospective studies, such as the selection bias of who was initially treated with photon RT. Given that all were treated with similar IMRT techniques, we feel this is unlikely to affect our results in any pronounced fashion. Another limitation is the lack of correlative toxicity and quality-of-life outcomes to pair with our dosimetric analysis. Correspondingly, we provided dosimetric endpoints that have been shown to correlate with toxicity within the Discussion and compared these to our results. No studies to date have reported outcomes for RPS treated with pre-operative ultra-hypofractionated IMPT thus far, and we are planning a follow-up study using normal tissue complication probability (NTCP) calculations to further investigate whether the dosimetric differences shown herein may correlate with clinical outcomes.

Our institution is in the process of opening a single-arm Phase II trial evaluating the use of pre-operative ultra-hypofractionated IMPT for RPS. Table 5 outlines ongoing trials utilizing pre-operative hypofractionated and/or particle irradiation for RPS.

## 5. Conclusions

Ultra-hypofractionated pre-operative proton therapy maintained target coverage while significantly reducing the dose to nearby organs at risk and integral dose compared to photon irradiation for patients with retroperitoneal sarcoma. Further investigation is warranted to validate these dosimetric findings and potential clinical benefits in the management of retroperitoneal sarcoma.

## Figures and Tables

**Table 1:** Target dose and coverage parameters.

Target	Goal	Hard Constraint	Dose (GyE)	Max Point Dose (%)
GTV	100% of the volume to 100% of the dose	98% of the volume to 100% of the dose	25	108
CTV	98% of the volume to 100% of the dose	95% of the volume to 100% of the dose	25	108
CTV Boost	95% of the volume to 100% of the dose	90% of the volume to 100% of the dose	30	108

**Table 2:** Institutional normal tissue constraints.

Tissue	Constraint
Stomach	Max Dose < 30 GyE
Bowel	Max Dose < 30 GyE
Liver	Spare at least 700 cc < 15 GyE
Kidneys	V12 GyE < 33%
Spinal Canal	Max Dose < 25 GyE

**Table 3:** Normal tissue constraints derived from BED equivalents of recommendations published by Baldini et al. [31].

Tissue	Published Dose Constraint	5 Fraction BED Equivalent
Stomach	V45 ≤ 100%	V25.55 ≤ 100%
	V50 ≤ 50%	V27.90 ≤ 50%
	Max Dose < 56 Gy	Max Dose < 30.65 Gy
Duodenum	V45 ≤ 100%	V25.55 ≤ 100%
	V50 ≤ 50%	V27.90 ≤ 50%
	Max Dose < 56 Gy	Max Dose < 30.65 Gy
Bowel	V15 < 830 cc	V10.45 < 830 cc
	V45 ≤ 195 cc	V25.55 ≤ 195 cc
Liver	Mean Dose < 26 Gy	Mean Dose < 16.30 Gy
Kidney, if Both Remain	Mean Dose < 15 Gy	Mean Dose < 10.45 Gy
	V18 < 50%	V12.10 < 50%
Kidney, if 1 Resected	V18 < 15%	V12.10 < 15%
Femoral Head	V40 < 64%	V23.20 < 64%
	Max Dose < 50 Gy	Max Dose < 27.90 Gy
	Mean Dose < 37 Gy	Mean Dose < 21.75 Gy
Spinal Canal	Max Dose < 50 Gy	Max Dose < 27.90 Gy

Abbreviations: GyE: radiobiological Gy equivalent, GTV: gross tumor volume, CTV: clinical target volume, cc: cubic centimeter, BED: 5-fraction biologically effective dose equivalents derived from 28-fraction constraints published by Baldini et al. [31].

**Table 4:** Target coverage, OAR doses, and integral dose, comparing IMRT and IMPT.

Target/OAR	Dosimetric Endpoint	IMRT		IMPT		*p*-Value
		Mean	St-Dev	Mean	St-Dev	
GTV	V25 GyE (%)	100	0.0	100	0.0	N/A
CTV	V25 GyE (%)	99.7	0.2	99.5	0.3	0.080
CTV Boost	V30 GyE (%)	100	0.0	100	0.0	N/A
PTV	V25 GyE (%)	96.5	0.7	97.6	1.2	0.003 *
PTV Boost	V30 GyE (%)	96.1	0.9	96.2	0.7	0.413
Stomach	V5 GyE (cc)	144.6	156.5	26.1	50.8	0.056
	V10 GyE (cc)	103.3	116.1	17.4	35.2	0.055
	V15 GyE (cc)	43.4	52.0	11.2	22.7	0.039 *
	V20 GyE (cc)	16.8	28.2	6.7	13.3	0.082
	V25 GyE (cc)	4.9	9.9	1.1	1.7	0.163
	D50% (GyE)	7.4	7.3	1.6	3.8	0.022 *
	Max Dose (GyE)	17.9	10.3	11.6	12.8	0.024 *
	Mean Dose (GyE)	7.8	7.2	2.1	4.3	0.019 *
Duodenum	V5 GyE (cc)	45.3	27.8	31.8	27.1	0.056
	V10 GyE (cc)	39.7	27.9	29.1	25.6	0.083
	V15 GyE (cc)	34.2	27.0	27.0	24.6	0.093
	V20 GyE (cc)	27.2	24.8	24.5	23.4	0.051
	V25 GyE (cc)	14.4	20.4	13.3	21.4	0.235
	D50% (GyE)	17.6	8.8	13.2	12.5	0.095
	Max Dose (GyE)	27.3	3.1	26.8	3.1	0.105
	Mean Dose (GyE)	16.5	6.5	11.6	8.7	0.040 *
Bowel	V5 GyE (cc)	1552.5	1127.2	503.8	412.5	0.002 *
	V10 GyE (cc)	916.3	642.8	414.8	346.1	0.000 *
	V15 GyE (cc)	554.9	444.1	347.0	294.1	0.001 *
	V20 GyE (cc)	351.3	299.5	280.0	240.4	0.007 *
	V25 GyE (cc)	138.6	135.6	157.3	149.9	0.095
	D50% (GyE)	8.9	5.5	1.3	3.4	0.000 *
	Max Dose (GyE)	27.3	4.0	25.6	8.9	0.161
	Mean Dose (GyE)	10.6	4.8	5.0	3.8	0.000 *
Liver	V5 GyE (cc)	570.9	846.4	302.5	603.4	0.048 *
	V10 GyE (cc)	393.0	689.2	184.6	329.6	0.056
	V15 GyE (cc)	266.7	534.2	125.5	212.3	0.112
	V20 GyE (cc)	141.5	258.0	93.0	159.5	0.107
	V25 GyE (cc)	48.8	94.9	48.1	101.5	0.460
	D50% (GyE)	4.7	7.8	2.3	5.0	0.029 *
	Mean Dose (GyE)	5.1	7.2	2.9	4.9	0.016 *
Ipsilateral Kidney	V10 GyE (%)	36.8	29.2	23.1	20.8	0.020 *
	V12 GyE (%)	33.0	26.2	21.2	19.6	0.021 *
	D50% (GyE)	8.2	6.9	3.8	4.2	0.006 *
	Mean Dose (GyE)	8.9	6.8	6.1	5.2	0.006 *
Contralateral Kidney	V10 GyE (%)	20.6	26.4	0.1	0.3	0.018 *
	V12 GyE (%)	10.5	14.0	0.1	0.2	0.021 *
	D50% (GyE)	4.6	4.6	0.0	0.0	0.006 *
	Mean Dose (GyE)	4.7	4.5	0.2	0.3	0.006 *
Bilateral Kidneys	V10 GyE (%)	28.1	24.1	10.5	11.1	0.007 *
	V12 GyE (%)	20.7	17.1	9.7	10.4	0.007 *
	D50% (GyE)	5.1	4.8	0.2	0.2	0.005 *
	Mean Dose (GyE)	6.8	5.1	2.9	2.8	0.003 *
Ipsilateral Femoral Head	V23.2 GyE (%)	13.0	17.5	4.4	9.3	0.041 *
	V30 GyE (cc)	0.9	2.2	1.7	4.1	0.182
	Max Dose (GyE)	15.0	16.1	13.4	14.9	0.092
	Mean Dose (GyE)	8.2	9.7	4.1	5.8	0.058
Contralateral Femoral Head	V23.2 GyE (%)	0.0	0.0	0.0	0.0	N/A
	V30 GyE (cc)	0.0	0.0	0.0	0.0	N/A
	Max Dose (GyE)	5.8	7.1	0.1	0.1	0.050
	Mean Dose (GyE)	3.2	4.5	0.0	0.0	0.070
Bone	V5 GyE (cc)	1131.4	521.9	690.1	296.3	0.005 *
	V10 GyE (cc)	897.2	443.7	446.5	213.0	0.001 *
	V15 GyE (cc)	610.6	364.5	303.2	193.7	0.001 *
	V20 GyE (cc)	378.1	299.4	219.4	173.9	0.003 *
	V25 GyE (cc)	191.4	170.7	144.2	118.0	0.016 *
	Mean Dose (GyE)	15.2	1.9	9.3	2.6	0.000 *
Spinal Canal	Max Dose (GyE)	19.4	6.5	13.9	8.9	0.004 *
Skin	V12 GyE (%)	10.5	13.5	9.4	9.4	0.346
Body	Max Dose (GyE)	32.6	0.8	31.6	0.2	0.000 *
Body—CTV	Integral Dose (J)	22.0	9.3	10.2	4.3	0.000 *

Abbreviations: OAR: organ at risk, IMRT: intensity-modulated (photon) radiation therapy, IMPT: intensity-modulated proton therapy, St-Dev: standard deviation, GTV: gross tumor volume, CTV: clinical target volume, PTV: planning target volume, GyE: radiobiological Gy equivalent, cc: cubic centimeter, J: joule. * Considered statistically significant based on *p*-value < 0.050

**Table 5:** Summary of prospective trials utilizing hypofractionation and/or particle irradiation for retroperitoneal sarcomas.

Phase	Identifier	Sponsor	Date of Initiation	Estimated Study Completion Date	Last Update Posted	Recruitment Status	Estimated Enrollment	Arm(s)	Primary Endpoint	Trial Design
**Hypofractionated photon trials**										
II	NCT03972930	University of Wisconsin (USA)	June 2019	September 2027	September 2022	Recruiting	48	IMRT—60 Gy3–8 fx (most commonly 6 fx) (QOD)	2-year local control as determined by RECIST	Single arm trial enrolling soft tissue sarcomas deemed unresectable of any location
II	NCT05224934	Chinese Academy of Medical Sciences (China)	January 2022	December 2024	February 2022	Recruiting	28	SBRT—25–50 Gy5 fx	Perioperative complications within 1 wk post-op	Single arm trial investigating feasibility and perioperative complications of pre-op SBRT followed by surgery 1–2 months later
**Conventionally fractionated particle therapy trials**										
I/II	NCT01659203	Massachusetts General Hospital (USA)	December 2012	August 2025	September 2020	Recruiting	Phase I: 11	Phase I: IMRT/IMPT—50.4 GyE (SIB: 60.2–63.0 GyE)28 fx	Phase I: maximum tolerated dose	Separate cohorts of patients receiving pre-op IMRT and IMPT. Phase I portion of each cohort utilized dose escalation for the SIB from 60.2 to 63.0 GyE showing no dose limiting toxicities, after which enrollment began on Phase II portion for each cohort
							Phase II: 60	Phase II: IMRT/IMPT—50.4 GyE (SIB: 63.0 GyE)28 fx	Phase II: local control	
III	NCT02838602	Hospices Civils de Lyon (France)	December 2017	December 2026	September 2021	Recruiting	250	Arm 1: Photon and/or proton RT—64.0–70.0 GyE * 32–35 fx	5-year progression free survival	Randomized trial comparing carbon vs photon and/or proton RT for radioresistant unresectable or resected with gross residual tumors, including chordomas, adenoid cystic head/neck cancers, and sarcomas
								Arm 2: Carbon—70.4–73.6 GyE * 16 fx (4 fxwk)		
**Hypofractionated particle therapy trials**										
II	NCT04219202	University Hospital Heidelberg (Germany)	May 2019	May 2024	June 2021	Recruiting	64	Arm 1: IMPT—39 GyE13 fx (6 fxwk)	Grade 3–5 toxicity	Randomized trial investigating safety and feasibility of hypofractionated, accelerated, pre-op RT based on grade 3-5 NCI-CTCAE toxicity and/or termination of planned therapy
								Arm 2: Carbon—39 GyE13 fx (6 fxwk)		
II	NCT05302570	Johns Hopkins University (USA)	December 2022 †	December 2027	July 2022	Not yet recruiting	45	IMPT—25 GyE (SIB: 30 GyE)5 daily fx	Grade 3–5 toxicity	Single arm trial evaluating safety and efficacy of hypofractionated pre-op proton therapy

Abbreviations: IMRT = intensity-modulated photon radiation therapy; fx = fractions; QOD = every other day; RECIST = Response Evaluation Criteria in Solid Tumours; SBRT = stereotactic body radiation therapy; wk = week; post-op = postoperative; pre-op = preoperative; IMPT = intensity-modulated proton therapy; GyE = Gray equivalent; SIB = simultaneous integrated boost to high-risk margin; RT = radiation therapy; vs = versus; NCI-CTCAE = National Cancer Institute Common Terminology Criteria for Adverse Events. * Recommended doses are dependent on histology, varying from 54.0–78.0 GyE for photon/proton arm and 60.8-73.6 GyE for carbon arm. Doses listed correspond to protocol recommendations for soft tissue sarcomas. † Estimated study start date.

## Data Availability

The data presented in this study are available on request from the corresponding author.
